# Supplementary material for: Active coacervate droplets as a model for membraneless organelles and protocells
Source: Nat Commun. 2020 Oct 14;11:5167. doi: 10.1038/s41467-020-18815-9 (PMC7560875; doi:10.1038/s41467-020-18815-9)
Supplement: Supplementary file 2 — Supplementary Information [file 41467_2020_18815_MOESM2_ESM.pdf]

Supporting Information for:

Active coacervate droplets as a model for membraneless organelles and protocells

Donau et al.

Supplementary Note 1: Determining anhydride formation.

The anhydride concentration could not be tracked via HPLC (intrinsic instability, peak overlap with precursor and low yield). Instead, an indirect quantification was used. Briefly, we reacted the anhydride with benzylamine at different time points (eg: 2, 4, … minutes) in the reaction cycle. Then, we measured the concentration of amide by HPLC. The addition of benzylamine is also stopping the formation of new anhydride because of the sudden raise in pH. A more detailed description of the quenching method will be published soon.

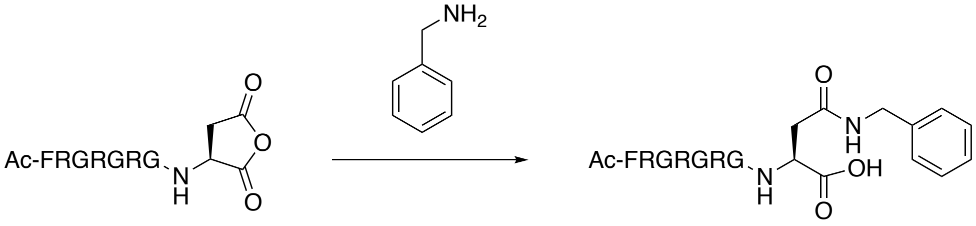

Supplementary Figure 1: Reaction scheme of the benzylamine quench.

To calculate the anhydride concentration from the benzylamine quenching method, it was approximated that abs_{220nm} (mono-amide) is the sum of abs_{220nm} (Ac-FRGRGRGD-OH) and abs_{220nm} (benzylamine). After calibration of benzylamine and Ac-FRGRGRGD-OH, we correlated the obtained integrals from the HPLC to [anhydride] and plotted the resulting concentration over time.

Supplementary Note 2: Full reaction scheme and all reaction described in the kinetic model.

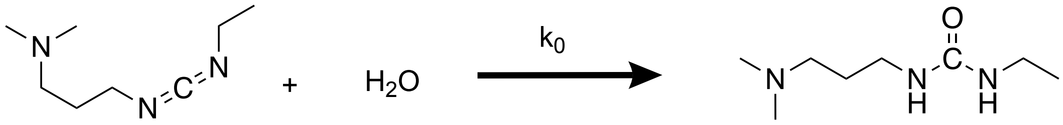

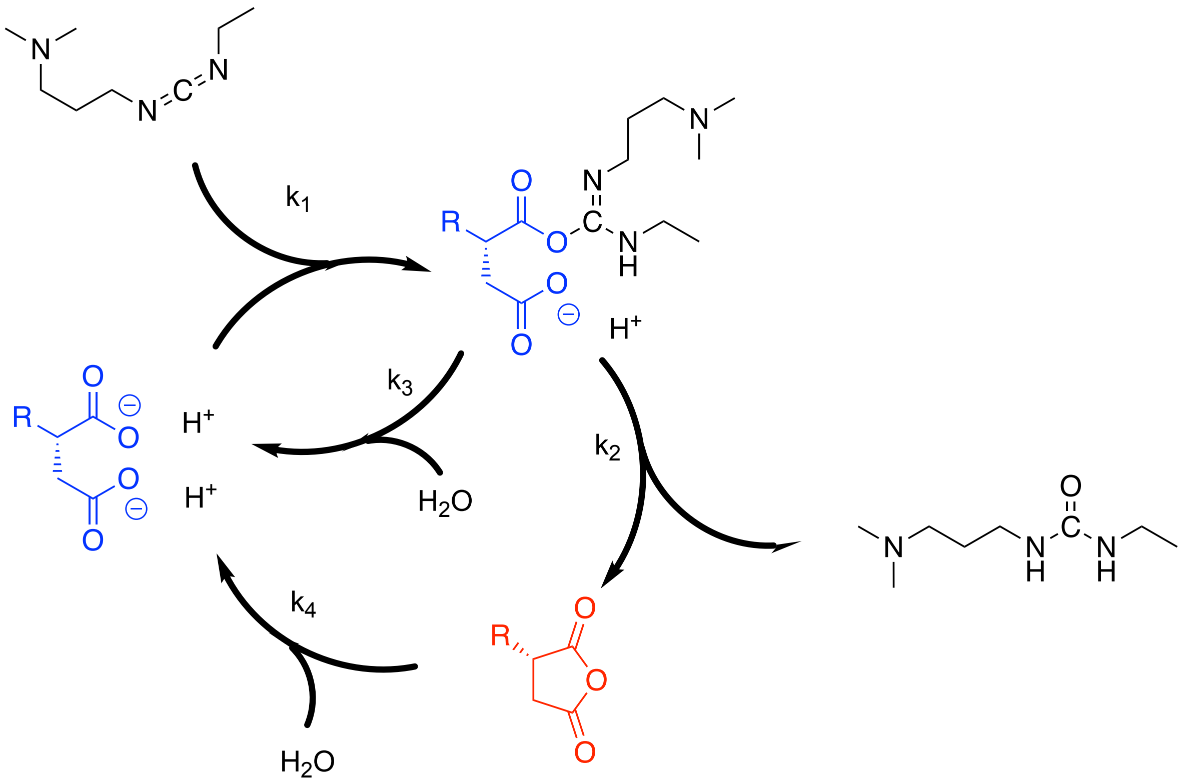

Supplementary Figure 2: All reactions taking place simultaneously in our reaction cycle.

The rate constants (k) refer to the rate constants used in our reaction model. kinetic model was written using Matlab in which the reactions below were described. The concentrations of each reactant were calculated for every second in the cycle.

Reaction 0 (k_0)

The direct hydrolysis of carbodiimide with a first order rate constant was set to 0. As this reaction is so slow in comparison to Reaction 1 (see below), it is irrelevant in the experiments. The consumption of EDC by poly(U) is neglectable.

Reaction 1 (k_1)

Formation of O-acylisourea by reaction with EDC. The rate constant was determined for the precursor by HPLC, by monitoring the EDC consumption.

Reaction 2 (k_2)

Formation of anhydride with a first order rate constant. This rate constant could not be determined because the O-acylisourea was never observed. It was set to be twice the rate of k_1.

Reaction 3 (k_3)

Direct hydrolysis of O-acylisourea (unwanted side reaction). This reaction rate could not be obtained because the O-acylisourea was not observed and so k_3 was set to zero times the rate of k_1.

Reaction 4 (k_4)

Hydrolysis of anhydride proceeded with a (pseudo)-first order rate as determined by HPLC.

Supplementary Table 1: Other peptide sequences synthesized for coacervation with poly-U.

Peptide sequence	*Observation*	*Explanation*
Fmoc-RGRGD-OH	EDC-induced turbidity, resulting in precipitation. No refueling possible.	*EDC-induced precipitation results in loss of peptide in solution.*
Fmoc-RGRGRGD-OH	Turbidity induced without EDC. Needed salt to inhibit turbidity. With additional salt, two EDC-induced cycles were possible, but precipitation occurred.	*Fmoc-group and three Rs interact too strongly with RNA. Additional salt weakens the electrostatic interactions. EDC-induced precipitation results in loss of peptide in solution.*
Ac-RRD-OH	No turbidity with low [peptide] and high [EDC].	*Two Rs interact too weakly with RNA.*
Ac-RRRD-OH	Precipitation for low [peptide] and high [EDC].	*Not trackable via HPLC since precursor peak overlaps with EDC.*
Ac-FRRRGD-OH	Due to precipitation only 2-3 reversible cycles possible.	*Three Rs interact too strongly with RNA.*
Ac-FRRGD-OH	Turbidity evolution very slow and not following our reaction kinetics.	*Two Rs interact too weakly with RNA, and only limited contribution of F.*
Ac-FRGRGD-OH	No turbidity with high [EDC] at high [peptide].	*Two spaced Rs interact too weakly with RNA.*
Ac-FHHHGD-OH	Due to precipitation no refueling with EDC possible.	*EDC-induced turbidity results in loss of peptide.*
Ac-FKGKGKGD-OH	In presence of EDC, irreversible consumption of precursor.	*Amino group of K reacts with EDC.*

Supplementary Table 2: Rate constants used to fit the HPLC data with the kinetic model (see methods).

k_1	k_2	k_3	k_4
O-acylurea formation	Anhydride formation	O-acylurea hydrolysis	Anhydride hydrolysis
0.075 (M^{-1} x s^{-1})	0.150 (s^{-1})	0.000 (s^{-1})	0.018 (s^{-1})

Supplementary Table 3: Partitioning coefficients of functional RNA in dynamic droplets, 5 minutes after fuel addition.

**RNA**	*Hammerhead*	*SunY*	*Broccoli*
**Nucleotides (nr)**	44	187	113
**Structure**	Folded	Folded	Folded
**Partitioning**	13	37	22

Standard conditions with 27 mM EDC and 0.1-0.2 µM of the respective fluorescently tagged (Cy-dyes) RNA.

Supplementary Table 4: DNA primers used in this work. The bold sequence in the T7 promoter and clamp primer is complementary to the underlined sequence in the rest of the primers.

Primer	Sequence (5’ to 3’)
T7 Promoter and Clamp	GATCGATCTCGCCCGC**GAAATTAATACGACTCACTATA**
F30 Broccoli	TTGCCATGAATGATCCCGAAGGATCATCAGAGTATGTGG-<br>GAGCCCACACTCTACTCGACAGATACGAATATC<br>TGGACCCGACCGTCTCCCACATACACATGGCAACCC<br>TATAGTGAGTCGTATTAATTTC
Hammerhead	TGTGCCTTTCGTCCTCACGGACTCATCAGTTCAGCTCCCTATAGTGAGTCG-<br>TATTAATTTC
SunY	GATCCTGCATGTCACCATGCAGTTCAGACTATATCTTCAACTCTTAGAG<br>TTGTCTGCCGTTTCGGGTCGCTTGACCCTACTCCCTTACATTCATCAGGATAG<br>TCGTTAGGCATTTACAGCTACTGCTGATTTAGCACGGGATTGACTCAGTGAGTGT<br>TTCCCCGTTTAGGCAGATTTTCCCTATAGTGAGTCGTATTAATTTC

Supplementary Table 5: RNA sequences used in this work. The * indicates the ligation junction between the RNA and the Cy5-tagged pentamer.

RNA	Sequence (5’ to 3’)
Hammerhead	GGGAGCUGAACUGAUGAGUCCGUGAGGACGAAAGGCACA*CACAU-Cy5
F30 Broccoli	GGGUUGCCAUGUGUAUGUGGGAGACGGUCGGGUCCAGAUAUUCGUAUCUGUCGA<br>GUAGAGUGUGGGCUCCCACAUACUCUGAUGAUCCUUCGG-<br>GAUCAUUCAUGGCAA*CACAU-Cy5
SunY	GGGAAAAUCUGCCUAAACGGGGAAACACUCACUGAGUCAAUCCCGUG<br>CUAAAUCAGCAGUAGCUGUAAAUGCCUAACGACUAUCCCUGAUGAAUGUAAGGG<br>AGUAGGGUCAAGCGACCCGAAACGGCAGACAACUCUAAGAGUUGAAGA<br>UAUAGUCUGAACUGCAUGGUGACAUGCAGGAUC*CACAU-Cy5
Cy5-Tag	CACAU-Cy5

Supplementary Table 6: Splints used in this work.

Splint	Sequence (5’ to 3’)
Hammerhead	ATGTGTGTGCCTTTC
F30 Broccoli	ATGTGTTGCCATGAA
SunY	ATGTGGATCCTGCAT

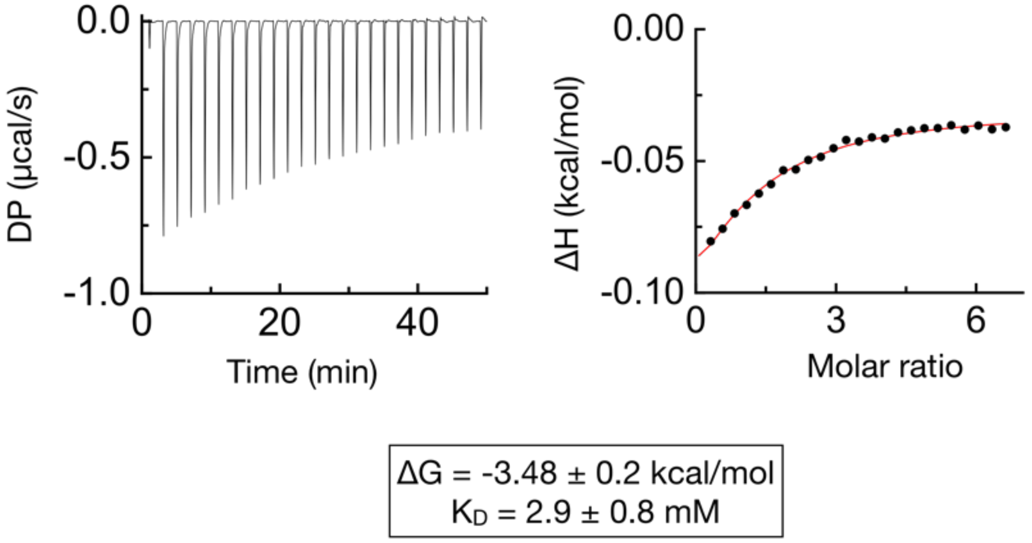

Supplementary Figure 3: Differential power and change in enthalpy measured by ITC for the interaction between precursor and RNA (poly-U). The resulting data (blank subtracted) were fitted assuming a 1:1 stoichiometry. Errors show the standard deviation from the average (N=3). For conditions, see methods.

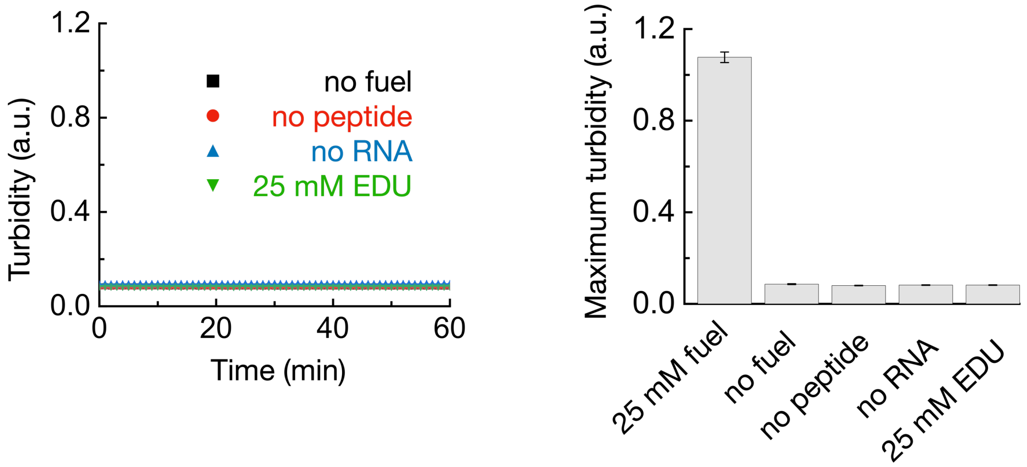

Supplementary Figure 4: Absorbance of 600 nm of light as a measure for turbidity of control experiments (not blank subtracted) at standard conditions. All error bars show the standard deviation from the average (N=3). We performed control experiments to demonstrate that our droplets are fuel-driven. When we added fuel to the same solution but either without RNA or without peptide, no turbidity was observed. Addition of a batch of waste as opposed to fuel also did not induce turbidity. The latter controls demonstrate that the droplets and their transient turbidity are a result of the fuel, RNA and peptide combined.

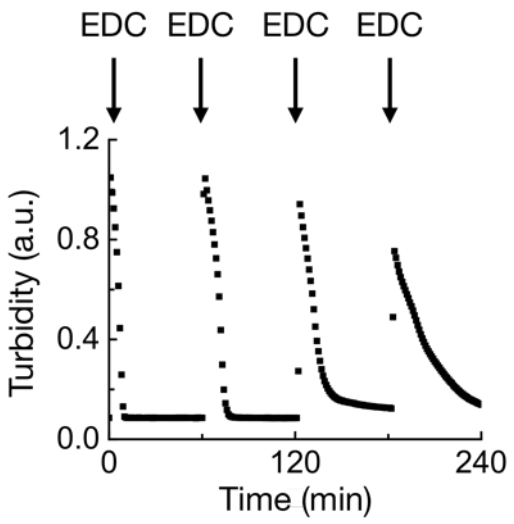

Supplementary Figure 5: Absorbance of 600 nm of light as a measure for the refueling of the chemical reaction network with additional batches of fuel (not blank subtracted). Standard conditions with 15 mM EDC. Re-addition of a batch of fuel triggers coacervation with similar turbidity and lifetime. However, after the second cycle, the system shows fatigue leading to lower turbidity and longer lifetimes. This observation can be attributed to the urea waste (EDU) which is accumulated during the cycle because of the consumption of EDC. EDU is known to screen charges of low molecular weight compounds and macromolecules which affects coacervation.

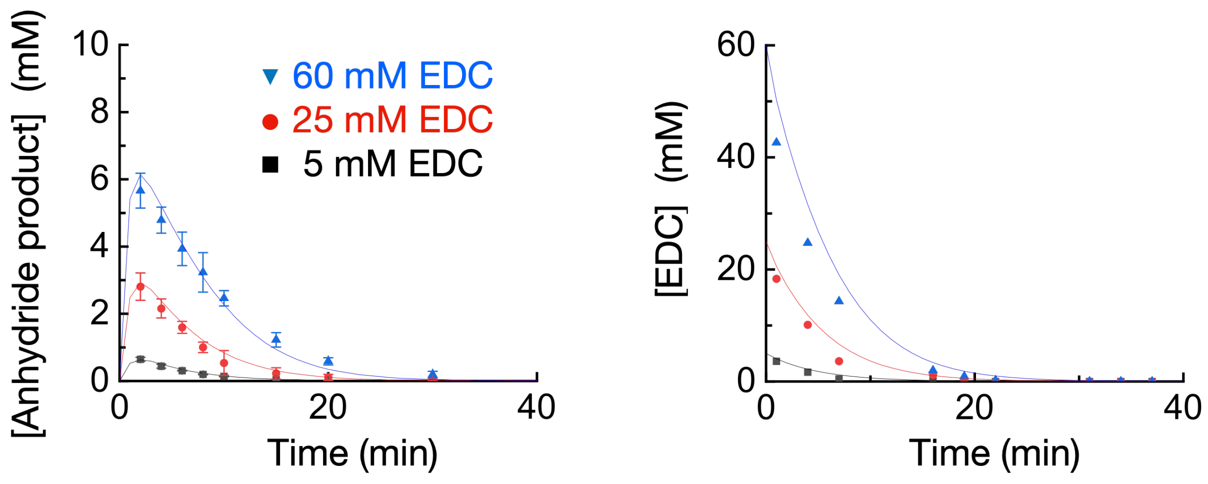

Supplementary Figure 6: Anhydride product and EDC concentrations from HPLC experiments. Standard conditions with 5 mM EDC (no droplets), 25 mM EDC (dynamic droplets) and 60 mM EDC (metastable droplets). All error bars show the standard deviation from the average (N=3). EDC traces are not in triplicates but from 3 different samples (1-16-31 min, 4-19-34 min, 7-22-37 min). Lines represent fitting plots from our kinetic model.

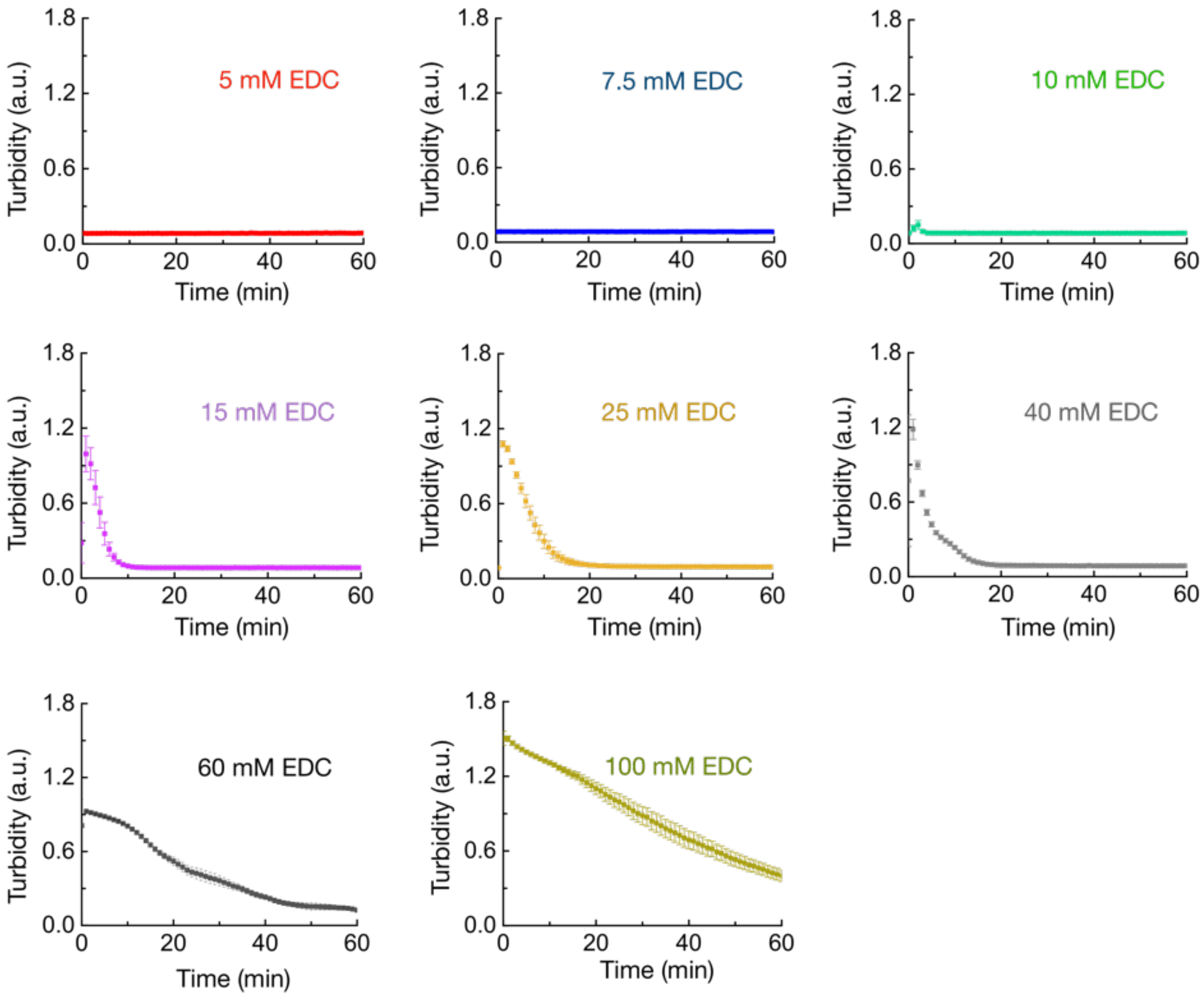

Supplementary Figure 7: Absorbance of 600 nm of light as a measure for turbidity for standard conditions with 4.1 mM poly-U and various fuel concentrations. Threshold value for dynamic droplets: absorbance < 0.1 a.u. (without blank subtraction) after 30 min. All error bars show the standard deviation from the average (N=3).

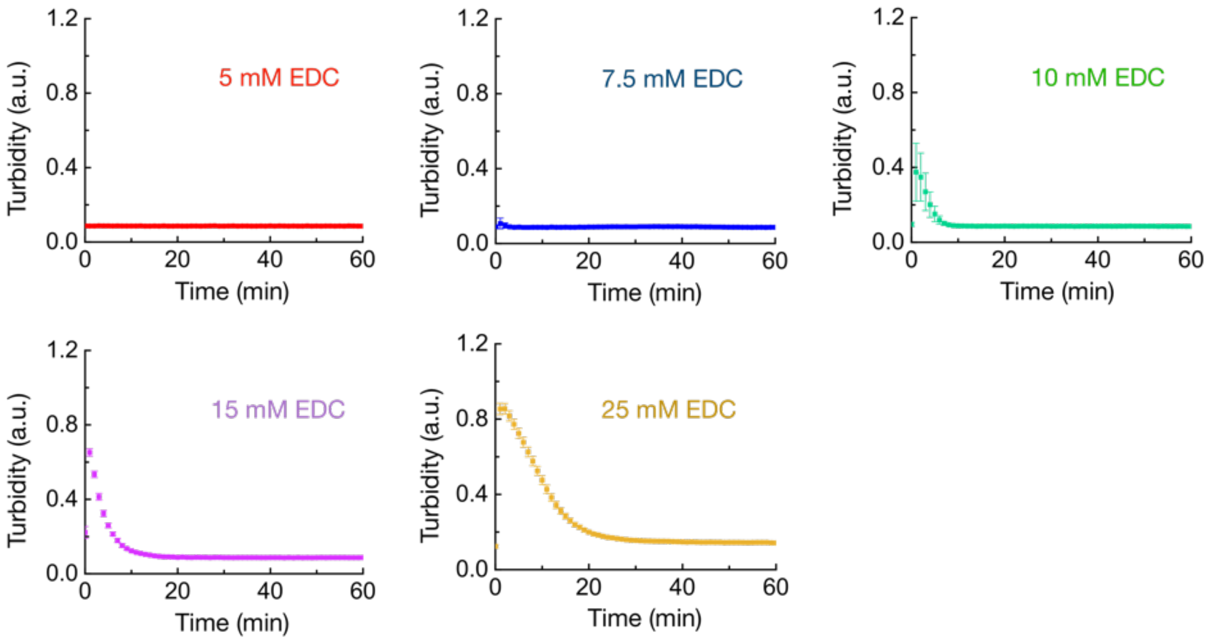

Supplementary Figure 8: Absorbance of 600 nm of light as a measure for turbidity for standard conditions with 1.4 mM poly-U and various fuel concentrations. Threshold value for dynamic droplets: absorbance < 0.1 a.u. (without blank subtraction) after 30 min. All error bars show the standard deviation from the average (N=3).

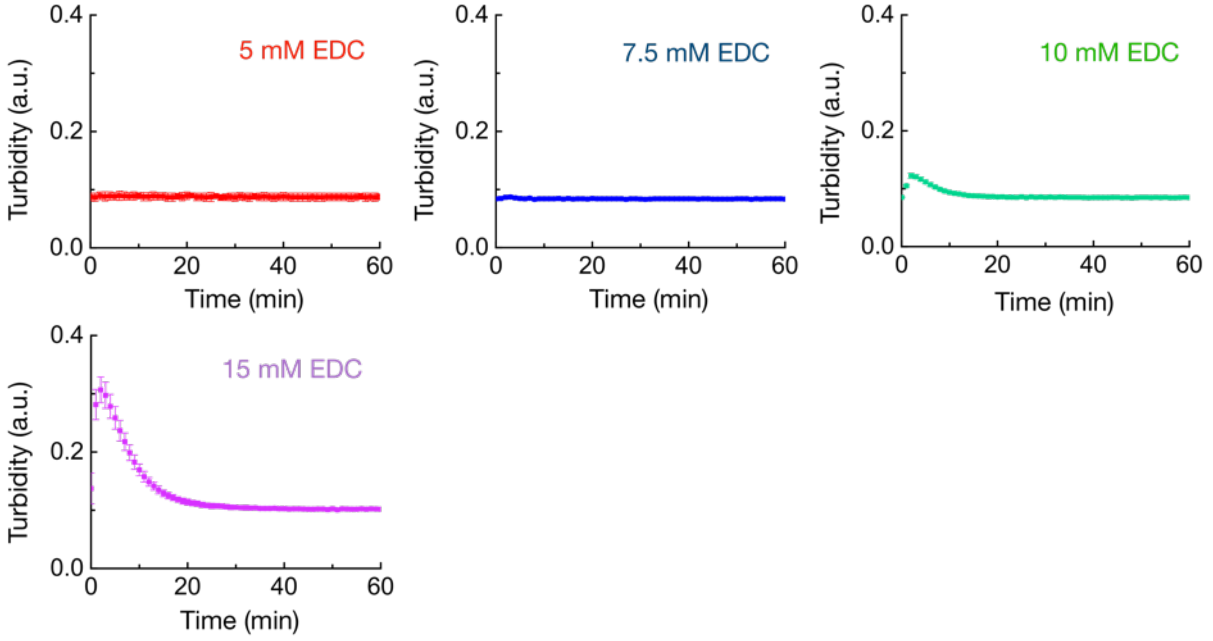

Supplementary Figure 9: Absorbance of 600 nm of light as a measure for turbidity for standard conditions with 0.3 mM poly-U and various fuel concentrations. Threshold value for dynamic droplets: absorbance < 0.1 a.u. (without blank subtraction) after 30 min. All error bars show the standard deviation from the average (N=3).

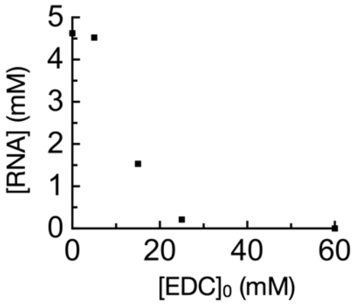

Supplementary Figure 10: Poly-U (RNA) concentration in the supernatant after centrifugation. 2 minutes after EDC addition. Standard conditions with various fuel concentrations.

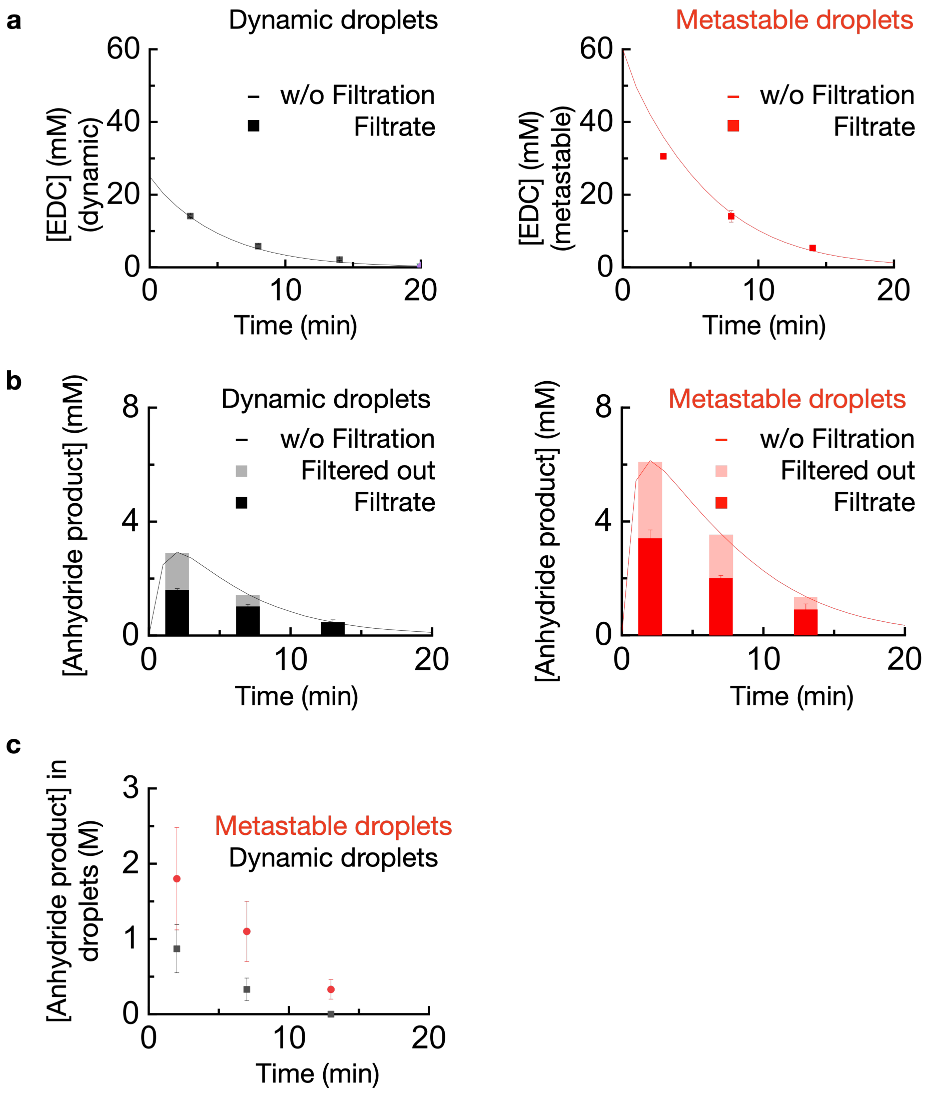

Supplementary Figure 11: EDC and anhydride product concentrations in the supernatant after filtration. **a**-**b**, EDC concentration (a) and anhydride product concentration (b) in the supernatant for dynamic (black) and metastable (red) droplets. **c**, Concentration of anhydride product in the droplet phase. Droplet volumes were estimated to be 0.3 ± 0.1 µL in a 200 µL sample (0.15 %). All error bars show the standard deviation from the average (N=3). Lines (w/o filtration) represent fitting plots from the kinetic model for the total amount of EDC and anhydride.

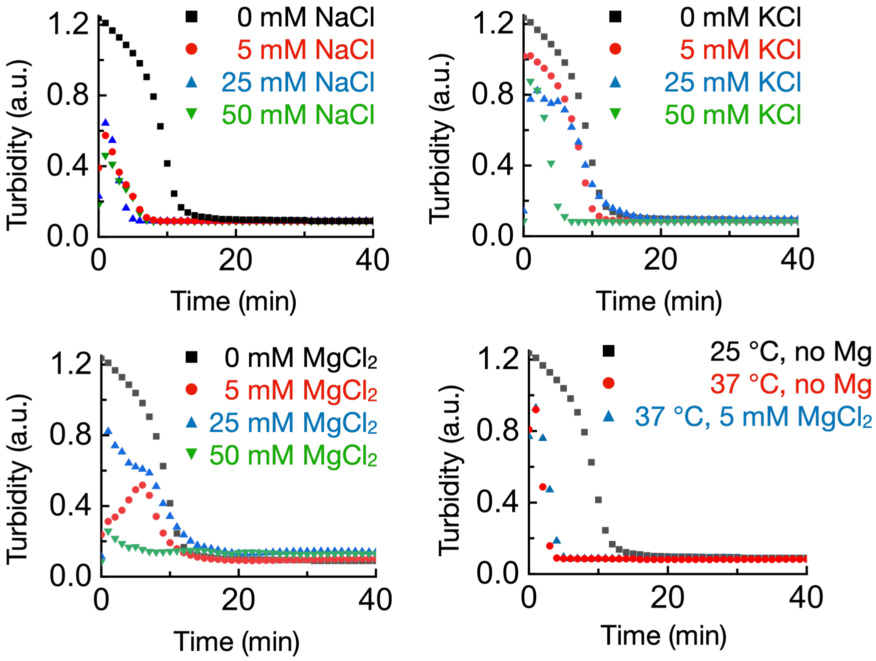

Supplementary Figure 12: Absorbance of 600 nm of light as a measure for turbidity for standard conditions and various salt concentrations or temperature.
