# Supplementary material for: Active coacervate droplets as a model for membraneless organelles and protocells
Source: Nat Commun. 2020 Oct 14;11:5167. doi: 10.1038/s41467-020-18815-9 (PMC7560875; doi:10.1038/s41467-020-18815-9)
Supplement: Supplementary file 3 — Descriptions of Additional Supplementary Files [file 41467_2020_18815_MOESM3_ESM.pdf]

Descriptions of Additional Supplementaty Files

Supplementary Movie 1

Description: Confocal microscopy timeseries of 23 mM precursor and 4.1 mM poly-U (Cy3-A15 hybridized) after the addition of 25 mM EDC. The complete cycle of dynamic droplets is shown.

Supplementary Movie 2

Description: Confocal microscopy timeseries of 23 mM precursor and 4.1 mM poly-U (Cy3-A15 hybridized) after the addition of 25 mM EDC. Vacuole formation in dynamic droplets is shown.

Supplementary Movie 3

Description: Confocal microscopy timeseries of 23 mM precursor and 4.1 mM poly-U (Cy3-A15 hybridized) after the addition of 25 mM EDC. Loss of mini-fragments of a single droplet is shown.

Supplementary Movie 4

Description: Confocal microscopy timeseries of 23 mM precursor and 4.1 mM poly-U (Cy3-A15 hybridized) after the addition of 25 mM EDC. Fragmentation of several droplets can be seen.
