# Supplementary material for: Active coacervate droplets as a model for membraneless organelles and protocells
Source: Nat Commun. 2020 Oct 14;11:5167. doi: 10.1038/s41467-020-18815-9 (PMC7560875; doi:10.1038/s41467-020-18815-9)
Supplement: Supplementary file 8 — Reporting Summary [file 41467_2020_18815_MOESM8_ESM.pdf]

nature research

Corresponding author(s): Job Boekhoven

Last updated by author(s): Aug 11, 2020

Reporting Summary

Nature Research wishes to improve the reproducibility of the work that we publish. This form provides structure for consistency and transparency in reporting. For further information on Nature Research policies, see our Editorial Policies and the Editorial Policy Checklist.

Statistics

For all statistical analyses, confirm that the following items are present in the figure legend, table legend, main text, or Methods section.

n/a	Confirmed	
☐	☒	The exact sample size (*n*) for each experimental group/condition, given as a discrete number and unit of measurement
☐	☒	A statement on whether measurements were taken from distinct samples or whether the same sample was measured repeatedly
☒	☐	The statistical test(s) used AND whether they are one- or two-sided<br>*Only common tests should be described solely by name; describe more complex techniques in the Methods section.*
☒	☐	A description of all covariates tested
☒	☐	A description of any assumptions or corrections, such as tests of normality and adjustment for multiple comparisons
☐	☒	A full description of the statistical parameters including central tendency (e.g. means) or other basic estimates (e.g. regression coefficient) AND variation (e.g. standard deviation) or associated estimates of uncertainty (e.g. confidence intervals)
☒	☐	For null hypothesis testing, the test statistic (e.g. *F*, *t*, *r*) with confidence intervals, effect sizes, degrees of freedom and *P* value noted<br>*Give P values as exact values whenever suitable.*
☒	☐	For Bayesian analysis, information on the choice of priors and Markov chain Monte Carlo settings
☒	☐	For hierarchical and complex designs, identification of the appropriate level for tests and full reporting of outcomes
☒	☐	Estimates of effect sizes (e.g. Cohen's *d*, Pearson's *r*), indicating how they were calculated

Our web collection on statistics for biologists contains articles on many of the points above.

Software and code

Policy information about availability of computer code

Data collection	Chromeleon (HPLC, v7.2 SR4), SkanIt (UV-Vis, v6.0.1), SpectraManager (Fluorescence, v2.13.00), Leica LAS AF (Confocal, v2.6.3.8173), MATLAB (kinetic model, v9.7.0), MicroCal PEAQ-ITC Analysis software (ITC, v1.22), OligoCalc (Functional RNA, v3.27)
Data analysis	Excel (v16.16.24), Fiji (v2.0)

For manuscripts utilizing custom algorithms or software that are central to the research but not yet described in published literature, software must be made available to editors and reviewers. We strongly encourage code deposition in a community repository (e.g. GitHub). See the Nature Research guidelines for submitting code & software for further information.

Data

Policy information about availability of data

All manuscripts must include a data availability statement. This statement should provide the following information, where applicable:

- Accession codes, unique identifiers, or web links for publicly available datasets
- A list of figures that have associated raw data
- A description of any restrictions on data availability

Data is available upon request.

Field-specific reporting

Life sciences study design

All studies must disclose on these points even when the disclosure is negative.

Sample size	Given the fact that the peptide was synthesized manually, we needed to work with lower volumes. Sample size was assumed to follow the volumes of the calibrated Eppendorf pipettes. 600 µL sample size for HPLC experiments (minimum volume required for the needle to inject). 200 µL sample size for the benzylamine quench (as the reaction is quenched, and convection does not play a role anymore, we worked with inlets to reduce amount of peptide needed). 150-200 µL sample size for fluorescence experiments (minimum volume required by the instrument). 50 µL sample size for confocal experiments were sufficient as the sample was not directly measured, but 5 µL were taken and imaged at a time.
Data exclusions	Data was not excluded.
Replication	Identical samples were prepared from the same stock solutions and the experiments were conducted for all the samples separately. All findings could be replicated.
Randomization	Not relevant for our study because samples were not allocated into groups.
Blinding	Not relevant to our study because not worked with participants.

Reporting for specific materials, systems and methods

We require information from authors about some types of materials, experimental systems and methods used in many studies. Here, indicate whether each material, system or method listed is relevant to your study. If you are not sure if a list item applies to your research, read the appropriate section before selecting a response.

Materials & experimental systems

n/a	Involved in the study	
☒	☐	Antibodies
☒	☐	Eukaryotic cell lines
☒	☐	Palaeontology and archaeology
☒	☐	Animals and other organisms
☒	☐	Human research participants
☒	☐	Clinical data
☒	☐	Dual use research of concern

Methods

n/a	Involved in the study	
☒	☐	ChIP-seq
☒	☐	Flow cytometry
☒	☐	MRI-based neuroimaging
